# Cutaneous manifestations of myelodysplastic syndrome: A systematic review

**DOI:** 10.1002/ski2.323

**Published:** 2024-02-01

**Authors:** Xiang Li Tan, Theodora Vatopoulou, Amana Siddique, Athena Kolovos, Ruth C. Lamb, Charlotte Fleming, Leila Ferguson, Victoria Akhras, Zainab Jiyad

**Affiliations:** ^1^ Department of Dermatology St George's University Hospitals NHS Foundation Trust London UK; ^2^ St Bartholomew's Hospital Barts Health NHS Trust London UK; ^3^ Department of Haematology St George's University Hospitals NHS Foundation Trust London UK; ^4^ St George's University of London London UK; ^5^ Population Health Research Institute St George's University of London London UK

## Abstract

Myelodysplastic syndrome (MDS) may present with specific skin lesions, such as leukaemia cutis, which is a well known poor prognostic marker of leukaemia with a high risk of acute leukaemic transformation. However, less is known regarding non‐specific cutaneous manifestations of MDS including the prevalence, types and their prognostic and therapeutic significance, which we aimed to determine through this systematic review. We searched electronic databases (PubMed, Medline and EMBASE) from inception up to 26 January 2023 for studies reporting cutaneous manifestations of MDS. Eighty eight articles (case reports *n* = 67, case series *n* = 21), consisting of 134 patients were identified. We identified 6 common cutaneous manifestations: neutrophilic dermatoses (*n* = 64), vasculitis (*n* = 21), granulomatous (*n* = 8), connective tissue disease (CTD) (*n* = 7; composed of dermatomyositis (*n* = 5), cutaneous lupus erythematosus (*n* = 1), and systemic sclerosis (*n* = 1)), panniculitis (*n* = 4), immunobullous (*n* = 1), and other (*n* = 29). Cutaneous features either occurred at time of MDS diagnosis in 25.3%, preceding the diagnosis in 34.7% (range 0.5–216 months), or after diagnosis in 40.0% (range 1–132 months). Prognosis was poor (40.2% death) with 34.1% progressing to acute myeloid leukaemia (AML). 50% of those with MDS who progressed to AML had neutrophilic dermatoses (*p* = 0.21). Myelodysplastic syndrome was fatal in 39.2% of neutrophilic dermatoses (median time from onset of cutaneous manifestation: 12 months), 50% of vasculitis (7.5 months), 62.5% of granulomatous (15.5 months) and 14.3% of CTD (7 months). Recognition of patterns of cutaneous features in MDS will improve early diagnosis and risk stratification according to subtype and associated prognosis.

## Graphical abstract

The prevalence and types of cutaneous manifestations in myelodysplastic syndrome (MDS) including its prognostic and therapeutic significance are unknown. Sweet syndrome is reported as the most common non‐specific cutaneous manifestation of MDS. We identified 6 common cutaneous manifestations: neutrophilic, vasculitis, granulomatous, connective tissue disease, panniculitis and immunobullous lesions. The majority which progressed to acute myeloid leukaemia (AML) were associated with neutrophilic dermatoses, however granulomatous skin lesions, had the poorest prognosis with the highest proportion progressing to AML. Limitations include publication bias. Recognition of cutaneous features in MDS will improve early diagnosis and risk stratification according to subtype and associated prognosis.

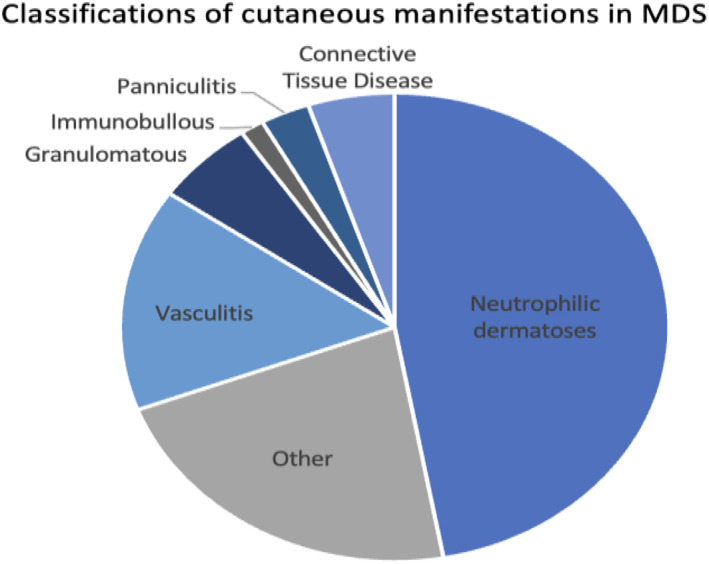

Myelodysplastic syndrome may present with specific skin lesions, such as leukaemia cutis, which is a well known poor prognostic marker of leukaemia with a high risk of acute leukaemic transformation.^1^, ^2^ However, less is known regarding non‐specific cutaneous manifestations of MDS including the prevalence, types and their prognostic and therapeutic significance, which we aimed to determine through this systematic review.

This study was conducted in accordance with the Preferred Reporting Items for Systematic Reviews and Meta‐Analyses guidelines (Table S1) and registered in PROSPERO (CRD42021293140). We searched electronic databases (PubMed, Medline and EMBASE) from inception up to 26 January 2023, using Medical Subject Headings and free text terms of the following concepts: 1) “myelodysplastic syndrome” OR “myelodysplastic myeloproliferative disease” and 2) “skin”, “cutaneous” or “dermatology”. Titles and abstracts were screened for initial eligibility following which full text publications were retrieved and assessed independently and in duplicate using the complete eligibility criteria in a standardised manner (XLT, and ZJ, AS, or AK). Discrepancies were resolved through discussions and involvement of haematology consultant (TV) where appropriate. All studies reporting cutaneous manifestations of MDS in adults aged ≥18 were included. Nomenclature of MDS was based on the revised 2016 World Health Organisation (WHO) classification of myeloid neoplasm.^2^, ^3^ Previous WHO classifications of MDS since first introduced in 2001 were also used for terms such as refractory anaemia, refractory anaemia with ring sideroblasts, and refractory anaemia with excess blasts. Haematological malignancies which are no longer classified as a subtype of MDS from the French‐American‐British (FAB) 1982 including chronic myelomonocytic leukemia and refractory anaemia with excess blasts in transformation were excluded. Unspecified classification of MDS published before the 2001 WHO classification was excluded. The search was limited to English language.

Eighty eight articles (case reports *n* = 67, case series *n* = 21), consisting of 134 patients were included (Figure S1). The detailed characteristics of the individual studies and outcomes are presented in Table S2 and summarised in Table 1. We identified 6 common cutaneous manifestations (Figure 1): neutrophilic dermatoses (*n* = 64), vasculitis (*n* = 21), granulomatous (*n* = 8), CTD (total *n* = 7; composed of dermatomyositis (*n* = 5), cutaneous lupus erythematosus (*n* = 1), and systemic sclerosis (*n* = 1)), panniculitis (*n* = 4), immunobullous (*n* = 1), and other (*n* = 29). Cutaneous features either occurred at time of MDS diagnosis in 25.3%, preceding the diagnosis in 34.7% (range 0.5–216 months), or after diagnosis in 40.0% (range 1–132 months). However no significant temporal associations were found. Prognosis was poor (40.2% death) with 34.1% progressing to AML, equivalent to intermediate risk MDS based on the WHO prognostic scoring system risk group. 50% of those with MDS who progressed to AML had neutrophilic dermatoses (*p* = 0.21). Myelodysplastic syndrome was fatal in 39.2% of neutrophilic dermatoses (median time from onset of cutaneous manifestation to death: 12 months), 50% of vasculitis (7.5 months), 62.5% of granulomatous (15.5 months) and 14.3% of CTD (7 months) leading to death. Median survival of MDS is reported to range from 0.8 to 8.8 years depending on severity based on the Revised International Prognostic Scoring Sytem.^2^ Quality of the evidence assessed by (XLT and ZJ) using the 20‐item Quality Appraisal Checklist for Case Series Studies, developed by the Institute of Health Economics using the Delphi method^4^ deemed all studies to be of low quality due to the nature of case reports and series (Table S3).

**TABLE 1:** Baseline characteristics of patients with myelodysplastic syndrome (MDS) and cutaneous manifestations.

	Total^a^ (*n* = 134)	Neutrophilic (*n* = 64)	Vasculitis (*n* = 21)	Granulomatous (*n* = 8)	CTD (*n* = 7)	Panniculitis (*n* = 4)	Immunobullous (*n* = 1)	Other (*n* = 29)
Age, years (SD)	61.0 (15.4)	58.9 (16.1)	57.9 (16.4)	70.9 (4.7)	58.1 (16.1)	64 (1.41)	67	62.9 (18)
Male, *n* (%)	95 (74.6)	43 (74.1)	13 (61.9)	6 (75)	4 (57.1)	4 (100)	1 (100)	24 (82.8)
Female, *n* (%)	33 (25.4)	15 (25.9)	8 (38.1)	2 (25)	3 (42.9)	0	0	5 (16.7)
**MDS subtype at diagnosis**, n								
MDS with unilineage dysplasia	27	10	5	1	2	1	0	8
MDS with ring sideroblasts	5	3	1	0	0	0	0	1
MDS with multilineage dysplasia	12	4	1	2	3	2	0	1
MDS with excess of blasts	23	10	7	0	1	0	1	4
MDS with excess of blasts type 1	8	4	1	0	0	0	0	3
MDS with excess of blasts type 2	9	7	0	1	0	0	0	1
MDS unclassifiable	3	1	1	0	1	0	0	0
MDS unspecified	36	18	2	4	0	1	0	11
**Prognosis (*n* = 102)**								
Survival, *n* (%)	59 (59)	31 (60.8)	5 (50)	3 (27.5)	6 (85.7)	3 (100)	1 (100)	10 (52.7)
Death, *n* (%) (including AML)	41 (41)	20 (39.2)	5 (50)	5 (62.5)	1 (14.3)	0	0	9 (47.3)
AML, n	14	7	1	3	0	0	0	2
Time to death (from skin), median months	9.5	12	7.5	13	7	NA	NA	11
**Onset** ^**b**^ **(*n* = 97)**								
Before, *n* (mean months)	33 (12.1)	15 (8.1)	6 (46.4)	4 (7.4)	1 (7)	2 (9)	0	9 (8.8)
At presentation, n	24	10	2	1	3	0	0	2
After, *n* (mean months)	38 (20.6)	16 (23.4)	3 (47.3)	2 (48)	2 (7.5)	0	1 (3)	9 (13.4)

Abbreviations: AML, acute myeloid leukaemia; CTD, connective tissue disease; MDS, myelodysplastic syndrome; NA, not applicable; SD, standard deviation.

^a^ Case series with summarised data where it was not possible to ascertain individual patient demographics, MDS type and associated cutaneous features are only included in the ‘Total’ count.

^b^ Onset: in relation to MDS diagnosis.

**FIGURE 1:**
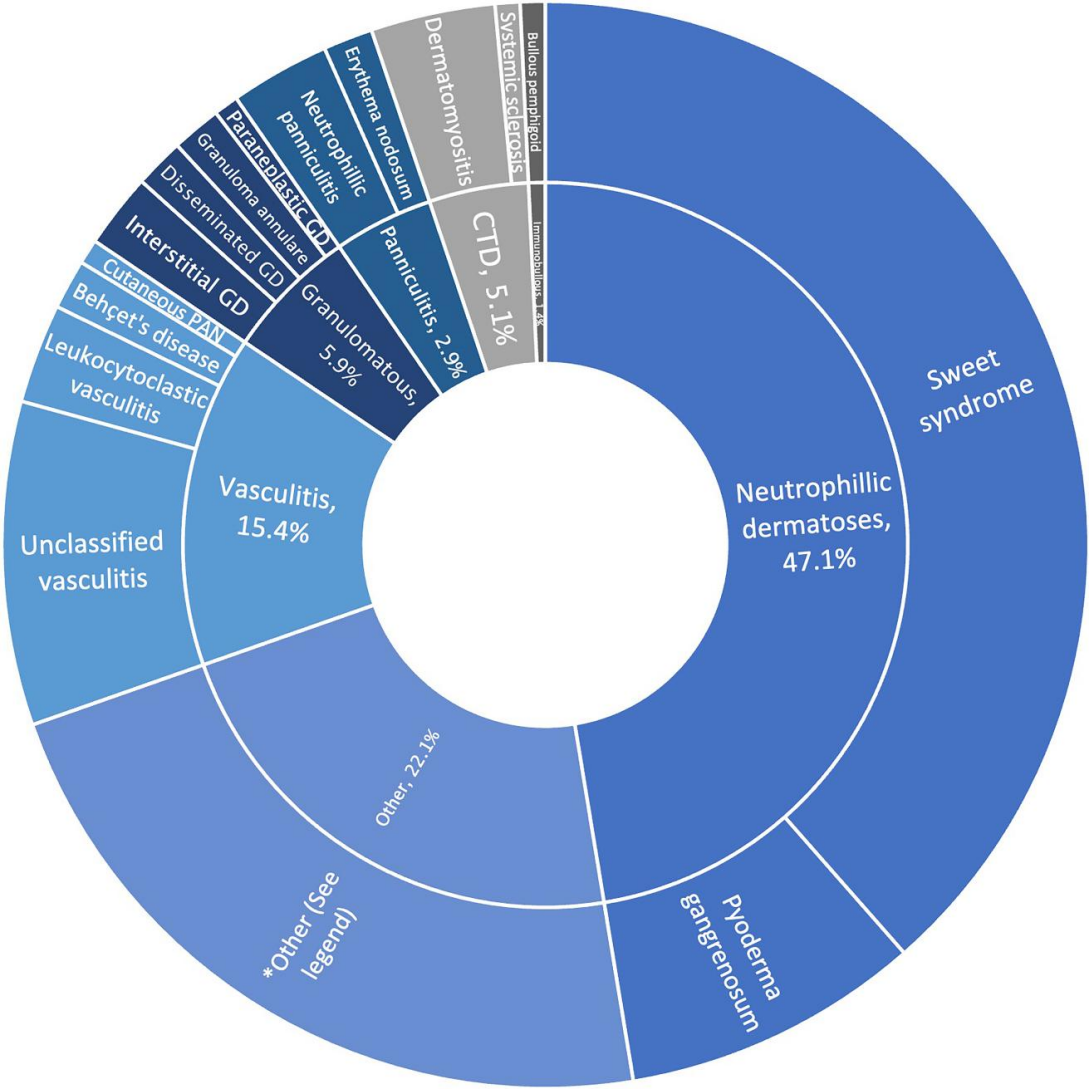
Classifications of cutaneous manifestations in myelodysplastic syndrome (MDS). CTD, connective tissue disease; GD, granulomatous dermatosis. *Other consists of: dermatofibroma, erosive pustular dermatosis, focal acantholytic dyskeratosis, granulolytic sarcoma, langerhans cell histiocytosis, lymphoma, papules, nodules, plaques, pruritic rash, sarcoid reaction, sarcoma, ulcers.

Neutrophilic dermatosis, in particular Sweet syndrome, was the most common cutaneous manifestations of MDS. These findings are consistent with a cohort study of 157 MDS patients which found a high prevalence of specific lesions and neutrophilic dermatosis.^5^ Histiocytoid sweet syndrome was reported to be more closely associated with MDS than neutrophilic sweet syndrome and often precedes diagnosis by 6 months.^6^ However, distinction between these types were not made in most included studies. A retrospective study of 82 MDS patients reported a high incidence of autoimmune manifestations,^7^ in line with our findings of vasculitis being the second most common manifestation, in addition to a sizeable portion of CTD and panniculitis. Granulomatous skin lesions have been reported to be associated with an aggressive clinical course of MDS.^8^ In keeping with this, patients with granulomatous lesions in our review had the highest mortality rate, of which 60% had progressed to AML prior to death.

Limitations include the proportion of case reports and small case series with associated risks of publication bias. There were inconsistencies in data presented leading to missing data, and insufficient data to enable meta‐analysis.

Recognition of patterns of cutaneous features in MDS will improve early diagnosis and risk stratification according to subtype and associated prognosis.

## AUTHOR CONTRIBUTIONS

**Xiang Li Tan**: Conceptualization (equal); Data curation (lead); Formal analysis (lead); Investigation (lead); Methodology (lead); Project administration (lead); Software (lead); Writing – original draft (lead); Writing – review & editing (lead). **Theodora Vatopoulou**: Conceptualization (equal); Methodology (equal); Supervision (equal); Validation (equal); Writing – original draft (equal); Writing – review & editing (equal). **Amana Siddique**: Data curation (equal); Formal analysis (equal); Investigation (equal); Writing – original draft (equal); Writing – review & editing (equal). **Athena Kolovos**: Data curation (equal); Formal analysis (equal); Investigation (equal); Writing – original draft (equal); Writing – review & editing (equal). **Ruth C. Lamb**: Conceptualization (equal); Supervision (equal); Validation (equal); Writing – original draft (equal); Writing – review & editing (equal). **Charlotte Fleming**: Conceptualization (equal); Supervision (equal); Validation (equal); Writing – original draft (equal); Writing – review & editing (equal). **Leila Ferguson**: Conceptualization (equal); Supervision (equal); Validation (equal); Writing – original draft (equal); Writing – review & editing (equal). **Victoria Akhras**: Conceptualization (equal); Supervision (equal); Validation (equal); Writing – original draft (equal); Writing – review & editing (equal). **Zainab Jiyad**: Conceptualization (lead); Data curation (equal); Formal analysis (equal); Investigation (equal); Methodology (equal); Project administration (equal); Supervision (lead); Validation (lead); Visualization (equal); Writing – original draft (lead); Writing – review & editing (lead).

## CONFLICT OF INTEREST STATEMENT

None to declare.

## ETHICS STATEMENT

The review was registered on PROSPERO and conducted in line with the PRISMA statement.

## Supporting information

Supplementary Material

Table S3

## Data Availability

The data from the included studies are available from the first author, XLT, upon reasonable request.
