# Supplementary material for: Cutaneous manifestations of myelodysplastic syndrome: A systematic review
Source: Skin Health Dis. 2024 Feb 1;4(2):e323. doi: 10.1002/ski2.323 (PMC10988661; doi:10.1002/ski2.323)
Supplement: Supplementary file 1 — Supplementary Material [file SKI2-4-e323-s002.docx]

Supplementary information

Figure S1. Flow diagram of study selection

Table S1. Preferred Reporting Items for Systematic Reviews and Meta-Analyses (PRISMA) Checklist

Table S2. Cutaneous Signs, Treatment Response and Clinical Outcomes of Patients with Myelodysplastic Syndrome

Table S3: Quality assessment of included studies (see excel document)

Appendix S1. References of included studies

**Figure S1.** Flow diagram of study selection

**
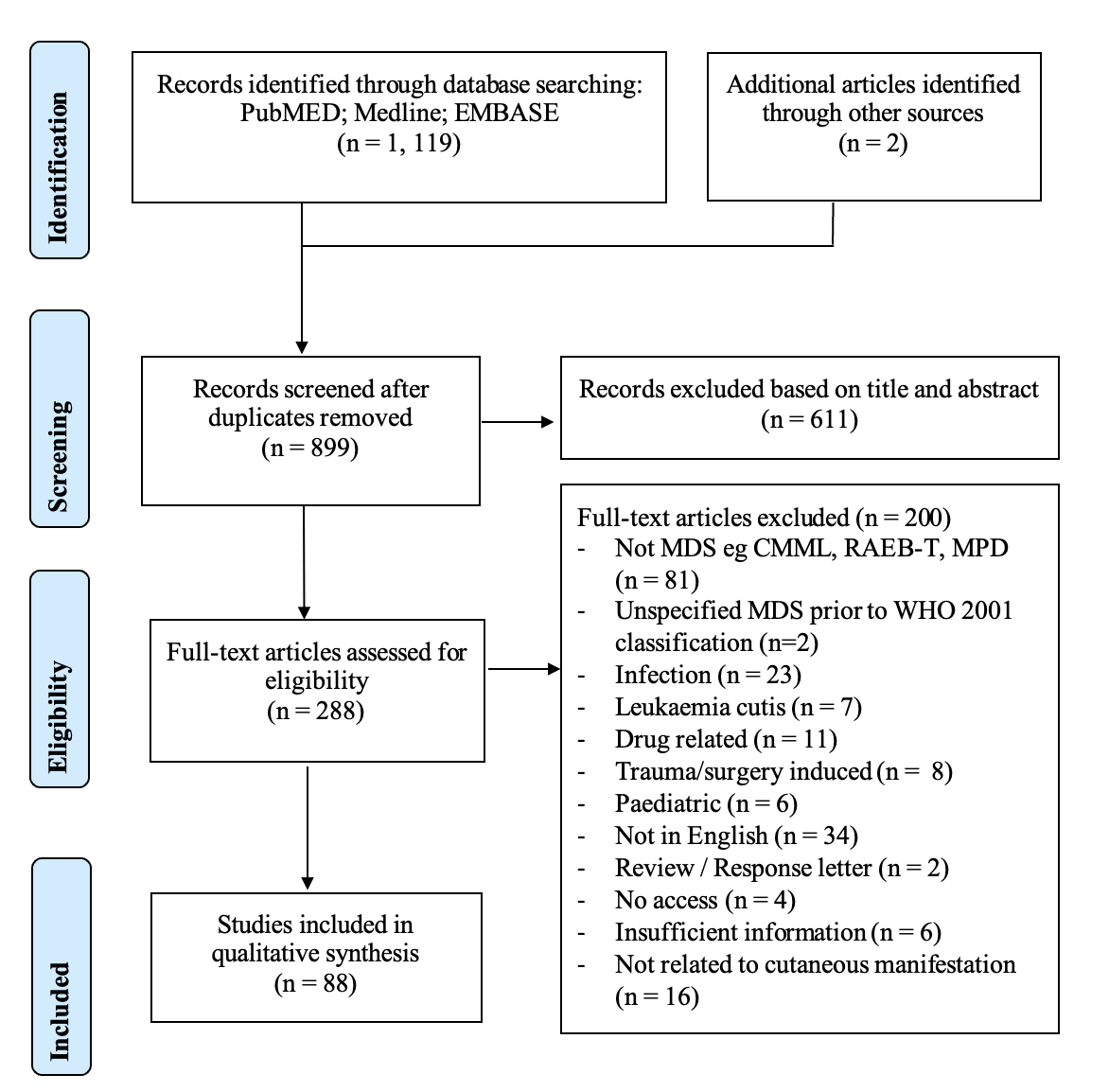
**

**Figure S1 Legend.** CMML, chronic myelomonocytic leukaemia; MDS, myelodysplastic syndrome; MPD, myeloproliferative neoplasm; RAEB-t, refractory anaemia with excess blast in transformation; WHO, world Health Organisation.

**Table S1.** Preferred Reporting Items for Systematic Reviews and Meta-Analyses (PRISMA) Checklist

| **Section/topic** | **#** | **Checklist item** | **Reported on page #** |
| --- | --- | --- | --- |
| **TITLE** | | | |
| Title | 1 | Identify the report as a systematic review, meta-analysis, or both. | Page 1 |
| **ABSTRACT** | | | |
| Structured summary | 2 | Provide a structured summary including, as applicable: background; objectives; data sources; study eligibility criteria, participants, and interventions; study appraisal and synthesis methods; results; limitations; conclusions and implications of key findings; systematic review registration number. | NA |
| **INTRODUCTION** | | | |
| Rationale | 3 | Describe the rationale for the review in the context of what is already known. | Page 4 |
| Objectives | 4 | Provide an explicit statement of questions being addressed with reference to participants, interventions, comparisons, outcomes, and study design (PICOS). | Page 4 |
| **METHODS** | | | |
| Protocol and registration | 5 | Indicate if a review protocol exists, if and where it can be accessed (e.g., Web address), and, if available, provide registration information including registration number. | Page 4 |
| Eligibility criteria | 6 | Specify study characteristics (e.g., PICOS, length of follow-up) and report characteristics (e.g., years considered, language, publication status) used as criteria for eligibility, giving rationale. | eMethods |
| Information sources | 7 | Describe all information sources (e.g., databases with dates of coverage, contact with study authors to identify additional studies) in the search and date last searched. | Page 4 |
| Search | 8 | Present full electronic search strategy for at least one database, including any limits used, such that it could be repeated. | eMethods |
| Study selection | 9 | State the process for selecting studies (i.e., screening, eligibility, included in systematic review, and, if applicable, included in the meta-analysis). | eMethods |
| Data collection process | 10 | Describe method of data extraction from reports (e.g., piloted forms, independently, in duplicate) and any processes for obtaining and confirming data from investigators. | eMethods |
| Data items | 11 | List and define all variables for which data were sought (e.g., PICOS, funding sources) and any assumptions and simplifications made. | eMethods |
| Risk of bias in individual studies | 12 | Describe methods used for assessing risk of bias of individual studies (including specification of whether this was done at the study or outcome level), and how this information is to be used in any data synthesis. | NA |
| Summary measures | 13 | State the principal summary measures (e.g., risk ratio, difference in means). | eMethods |
| Synthesis of results | 14 | Describe the methods of handling data and combining results of studies, if done, including measures of consistency (e.g., I^2^) for each meta-analysis. | NA |
| Risk of bias across studies | 15 | Specify any assessment of risk of bias that may affect the cumulative evidence (e.g., publication bias, selective reporting within studies). | NA |
| Additional analyses | 16 | Describe methods of additional analyses (e.g., sensitivity or subgroup analyses, meta-regression), if done, indicating which were pre-specified. | NA |
| **RESULTS** | | | |
| Study selection | 17 | Give numbers of studies screened, assessed for eligibility, and included in the review, with reasons for exclusions at each stage, ideally with a flow diagram. | eFigure 1 |
| Study characteristics | 18 | For each study, present characteristics for which data were extracted (e.g., study size, PICOS, follow-up period) and provide the citations. | eTable 1 |
| Risk of bias within studies | 19 | Present data on risk of bias of each study and, if available, any outcome level assessment (see item 12). | NA |
| Results of individual studies | 20 | For all outcomes considered (benefits or harms), present, for each study: (a) simple summary data for each intervention group (b) effect estimates and confidence intervals, ideally with a forest plot. | Table 1, eTable 1 |
| Synthesis of results | 21 | Present results of each meta-analysis done, including confidence intervals and measures of consistency. | NA |
| Risk of bias across studies | 22 | Present results of any assessment of risk of bias across studies (see Item 15). | NA |
| Additional analysis | 23 | Give results of additional analyses, if done (e.g., sensitivity or subgroup analyses, meta-regression [see Item 16]). | NA |
| **DISCUSSION** | | | |
| Summary of evidence | 24 | Summarize the main findings including the strength of evidence for each main outcome; consider their relevance to key groups (e.g., healthcare providers, users, and policy makers). | Page 4, 5 |
| Limitations | 25 | Discuss limitations at study and outcome level (e.g., risk of bias), and at review-level (e.g., incomplete retrieval of identified research, reporting bias). | Page 5 |
| Conclusions | 26 | Provide a general interpretation of the results in the context of other evidence, and implications for future research. | Page 5 |
| **FUNDING** | | | |
| Funding | 27 | Describe sources of funding for the systematic review and other support (e.g., supply of data); role of funders for the systematic review. | Title page |

**Table S2.** Cutaneous Signs, Treatment Response and Clinical Outcomes of Patients with Myelodysplastic Syndrome

| Study, Year | Age, Sex | MDS type | Onset^a^  (month) | Cutaneous Signs (n) | Treatment and Response | Clinical Outcomes n (%) Survival |
| --- | --- | --- | --- | --- | --- | --- |
| Case reports | |  |  |  |  |  |
| Antic, 2013 | 76, F | RAEB-1 | –6 | **Other/Erythematous firm plaques**  *Site: cheek* | For local radiotherapy, lost to f/u, refused | Lost to follow up |
| Arandes-Marcocci, 2015 | 80, M | RAEB-1 | –0.75 | **Other/Myelodysplasia cutis**  *Site: upper limb* | 5-azacitidine - cutaneous lesions almost disappeared. | 0 (0%) survival  *Cause of death:* haemorrhagic complications.18 months later |
| Ashida, 2006 | 53, M | RAEB | NA | **Neutrophilic/Sweet syndrome**  *Site:* *scrotum, face and oral cavity* | Prednisolone 60mg/day; combo chemo (aclarubicin and cytosine arabinoside), allogenic bone marrow stem cell transplantation (busulfan 8 mg/kg PO and fludarabine 180 mg/m^2^ IV); G-CSF 5 mg/kg/day. | 1 (100%) survival  Remission  Time not reported |
| Balin, 2011 | 71, M | Unspecified | 10 | **Granulomatous/interstitial granuloma annulare**  *Site: trunk, upper and lower extremities* | Hydrochloroquine sulfate 200mg BD – no improvement  Lenalidomide – skin improved after 6 weeks | 0 (0%) survival  *Cause of death:* AML, pneumonia, 12 weeks after presentation |
| Bhattacharjee, 2004 | 52, M | Unspecified | +1 | **Other/Multiple eruptive dermatofibromas**  *Site: abdomen, back, arms and legs* | NA | 1 (100%) survival  No change in lesions |
| Chamoun, 2018 | 76, M | Unspecified | NA | **Other/Violaceous plaques**  *Site:* *Abdomen* | CD123‐targeted therapy with SL‐401. | 1 (100%) survival  Remission |
| Chen, 2004 | 59, M | RAEB | 0 | **Panniculitis/neutrophilic panniculitis**  *Site:* *face, trunk, back, and extremities; whole body* | Systemic antibiotic (no improvement)  Prednisolone 40mg OD 0.5mg/kg (rapid improvement; reintroducted as intermittent fever persisted) | 0 (0%) survival  *Cause of death:* septic shock and pneumonia lung infection. (16 months later) |
| Choi, 2006 | 80, M | Unspecified | –5 | **Neutrophilic/Sweet syndrome**  *Site*: *trunk and extremities* | Prednisolone, dapsone 50mg OD | 1 (100%) survival  Stable, symptom recurred after treatment |
| de Arruda Camara, 2008 | 82, M | Unspecified | +11 | **Other/Sarcoma, nodules**  *Site: trunk, face, scalp and extremities* | Platelet transfusions | 0 (0%) survival  *Cause of death:* pulmonary haemorrhage and cardiovascular arrest six months after the diagnosis of granulocytic sarcoma |
| Delplanque, 2019 | 67F | Multilineage dysplasia | +12 | **CTD/CLE**  *Site: hands, thighs, breast and face* | hydroxychloroquine, topical and oral steroid | 1 (100%) survival  *Good response* |
| Farmakis, 2015 | 82, M | Unspecified | –6 | **Vasculitis/Leukocytoclastic vasculitis**  *Site: lower limbs* | RBC, filgrastim and epoetin alfa, methylpred 50mg OD | 0 (0%) survival  *Cause of death: progressive gangrene 3 months later* |
| Fein, 2000 | 74, M | Unspecified | –6 | **Vasculitis/Asymptomatic purpura**  *Site: forearm* | NA | NA |
| Goto, 2006 | 74, M | Unspecified | +24 | **Neutrophilic/PG**  *Site: lower limb, fingers* | Prednisolone 60mg OD, tapered down to 40mg OD (responded initially) | 0 (0%) survival  *Cause of death:* *patient died 3 months after admission* |
| Gubinelli, 2003 | 65, M | RAEB | +12 | **Neutrophilic/Sweet syndrome**  *Site: lower limbs, trunk, hands* | Prednisone (10 mg/day).+ EPO (10,000 UI 3 times/week) + dapsone 100 mg/day | 1 (100%) survival  *Remission* |
| Hagiwara, 2008 | 64, M | RAEB-2 | –12 | **Granulomatous/IGD**  *Site: leg trunk, face* | Prednisolone 30 mg/day, nicotinic acid amide 1.5 mg/day, doxycycline  hydrochloride 200 mg/day | 1 (100%) survival  Remission |
| Hattori 2003 | 69, M | Single lineage dysplasia | +4 | **Neutrophilic/Sweet syndrome**  *Site: legs* | NA | NA |
| Hojo, 2004 | 73, M | Single lineage dysplasia | +3 | **Other/Tender erythematous nodules**  *Site: abdomen, thighs, back* | Prednisolone at 40mg/day | 1 (100%) survival  *Good* |
| J Ten Oever, 2018 | 73, M | RAEB-2 | +12 | **Neutrophilic/Sweet syndrome (Histiocytoid)**  *Site: trunk, upper extremities* | Prednisone 70 mg/day, weaning dose and doxycycline 10/13 months. Recurred on tapering. | 0 (0%) survival  *Cause of death: bilateral pneumonia / progressed to myeloid sarcoma* |
| Kakaletsis, 2014 | 1 | RAEB | 0 | **Neutrophilic/Sweet syndrome – lymphocytic**  *Site: trunk, face, extremities* | Broad spectrum antibiotics and antifungals  Topical corticosteroids (resolved)  Azacitidine | NA |
| Kamimura, 2021 | 62, M | Single lineage dysplasia | –2 | **Neutrophilic/Sweet syndrome**  *Site: chest, upper arm* | oral prednisolone (20 mg/day) + treatment with corticosteroid pulse therapy, followed by azacitidine | 0 (0%) survival  *Cause of death: MDS progressed* |
| Kawakami, 2008 | 76, M | Unspecified | +14 | **Others/cutaneous extramedullary haematopoiesis mass**  *Site:* *scalp, chest, abdomen, back, and extremities* | Prednisolone + hydroxyurea (500 mg/d) + methylprednisolone- pulse regimen consisting of 1000 mg for 3 days, 350 mg/d of cyclosporine | 0 (0%) survival  *Cause of death:* *Developed a blast crisis, renal failure* |
| Khodadad, 2005 | 30, M | RAEB-2 | –1 | **Neutrophilic/Sweet syndrome**  *Site: face, chest, upper and lower limbs* | Cloxacillin and ceftazidime (resistant)  Prednisolone 60mg/day (resolved), discharged on 20mg/day | 0 (0%) survival  *Cause of death:* *minimally differentiated acute myelogenous leukemia (AML-M0) 1 year later* |
| Kim, 2020 | 48, F | RAEB-1 | 0 | **Neutrophilic/PG**  *Site: forehead* | 2/52 IV methylprednisolone (80 mg/day) -> skin lesions improved dramatically after 2 weeks.  Chemotherapy with methylprednisolone (20 mg/day) | 1 (100%) survival  Remission  Good response |
| Komiya, 1990 | 54, M | RAEB | –2 | **Neutrophilic/Sweet syndrome**  *Site: face, neck, chest, and upper extremities* | Oral prednisone 20mg OD (improved) | 0 (0%) survival  *Cause of death:* Died 4 months after cutaneous features from multiple aspergillomas and fungal emboli in lungs/shock |
| Lee, 2011 | 67, M | RAEB | +3 | **Immunobullous/Bullous pemphigoid**  *Site:* *limbs, especially acral region* | Oral prednisolone 30 mg daily (0.5 mg /kg/day) and azathioprine 100 mg daily. | 1 (100%) survival  Remission  Rapid response |
| Lee, 2016 | 44, M | Multilineage dysplasia | 0 | **Vasculitis/Unclassified vasculitis**  *Site: lower legs* | IV methylprednisolone (1 mg/ kg) | 1 (100%) survival  Remission  Rapid response |
| Lerman, 2019 | 70, M | RAEB | 0 | **CTD/Dermatomyositis**  *Site:* *scalp; bilateral hands* | Chemotherapy: azacytidine and pevonedistat  Topical triamcinolone 0.1% and tacrolimus 0.1% ointment | 1 (100%) survival  Remission |
| Litvak, 2000 | 63, M | Unspecified | NA | **Neutrophilic/PG**  *Site: R shin* | Prednisolone 40mg daily, cyclosporine 100mg twice a day, whirlpool therapy and split thickness skin graft. | 1 (100%) survival  *Good response* |
| Martinelli, 2014 | 66, M | RAEB-2 | –24 | **Neutrophilic/Sweet syndrome**  *Site: upper limb, trunk, neck, face* | 1. IV methylprednisolone, colchicine,  indomethacin, dapsone, minocycline and  methotrexate. (refractory)  2.5-Aza (improvement from 2^nd^ cycle onwards) | 1 (100%) survival  *Remission* |
| Martinez-Garcia, 2020 | 54, M | Single lineage dysplasia | +24 | **Other/Xanthogranulomas**  *Site*: trunk | No treatment | 1 (100%) survival  Stable |
| Marullo, 1989 | 62, F | RAEB | –2 | **Neutrophilic/Sweet syndrome**  *Site: forehead, arms, thighs, legs* | RBC, amikacin 700mg OD, ceftriaxone 1gm/day for 1 week without any clinical improvement. Isoniazide 200mg OD 3 weeks (no response). Prednisolone 0.75mg/kg/day f | 0 (0%) survival  *Cause of death:Llisterial meningitis after 3 months* |
| Mégarbane, 2000 | 34, M | RAEB | –2 | **Neutrophilic/Sweet syndrome**  *Site:* *neck, face, legs, oral mucosa* | IV methyloped 1g, oral pred 1mg/kg OD.  Ticarcilin/clauvulanic acid and gentamicin (resolved)  Bone marrow transplant and cyclophosphadmie (good) Relapse after tapering. | 1 (100%) survival  Remission |
| Mizes, 2020 | 59, F | Unclassifiable | +3 | **Neutrophilic/Sweet syndrome**  *Site:* *lower extremities* | IV methylprednisolone + 6-week oral prednisone taper. | NA |
| Nakanishi, 2015 | 66, F | Multilineage dysplasia | –7 | **CTD/Dermatomyositis**  *Site:* *shoulders, elbows, knees and hips* | Oral prednisolone 40 mg OD for skin lesions  Supportive care with blood transfusion | 0 (0%) survival  *Cause of death:* respiratory failure (linked to NSIP flare-up) 53 days after her admission |
| Namba, 1999 | 50, F | RAEB | +12 to 24 | **Others/Nodules**  *Site: scalp; extremities* | 1. Transfusion and chemotherapy  2. Radiation and oral steroids, not effective. | 0 (0%) survival  *Cause of death:* Feb 1996 from MOF, AML |
| Nawata, 2017 | 26, M | Multilineage dysplasia | 0 | **CTD/Dermatomyositis**  *Site:* *extremities* | Methylprednisolone 1g/day, oral prednisolone (1 mg/kg/day), and cyclosporine A IV | 1 (100%) survival  Remission |
| Nifosì, 2001 | 38, M | Single lineage dysplasia | 0 | **Neutrophilic/Sweet syndrome**  *Site:* *limbs* | 1. Corticosteroid therapy, colchicine (ineffective)  2. Indomethacin 150 mg / day, substitutive therapy. | 1 (100%) survival  Remission |
| Nishie, 2002 | 63, M | Unspecified | –18 | **Neutrophilic/Sweet syndrome**  **Panniculitis/Erythema nodosum**  *Site:* *lower legs; face, neck, chest, upper extremities* | 1) Ibuprofen 600mg OD  2) 7/7 Oral prednisolone 20 mg OD | 1 (100%) survival  Stable |
| Nizery-Guermeur, 2015 | 77, M | RAEB-1 | +35 | **Other/Granulocytic sarcoma**  *Site: thigh and cheek* | Cloxacillin for 18 days, 1 g 3 times per day / After regression of GS: chemotherapy (3 rounds of cytarabine/mitoxantrone) for MDS | 1 (100%) survival  Remission |
| O'Donnell, 1995 | 58, F | RAEB | –0.5 | **Vasculitis/Urticarial papules and plaques**  *Site: knees (extensor), the heels, hand dorsum* | Prednisolone - Initial improvement in her leg plaques On tapering - developed new plaques on her face. | 0 (0%) survival  *Cause of death:* AML 3 months later, died 7 months after vasculitis |
| Palterer, 2017 | 78, F | Single lineage dysplasia | 0 | **CTD/Dermatomyositis**  *Site: Face, eyes, hands* | 1. IV methylprednisolone (1 g/OD) and high-dose IVIG (30 g OD)  2. Methotrexate 15 mg/weekly added when corticosteroids were tapered. | 1 (100%) survival  NA |
| Papaioannou, 2008 | 78, M | RAEB-2 | +3 | **Other/Generalised skin rush and pruritus**  *Site: scalp* | Decitabine 20 mg ⁄ m2 ⁄ d i.v. 5 d every 4 wk. - Skin and pruritis regressed after 4 cycles and remission after 2 further cycles | 1 (100%) survival  *Partial response* |
| Patsinakidis, 2014 | 73, M | Multilineage dysplasia | 0 | **Granulomatous/IGD**  *Site:* *thighs, lateral aspects of the trunk, both arms* | Topical corticosteroid for skin lesions  + 5-azacytidine for MDS | 1 (100%) survival  Remission |
| Peñas, 1994 | 85, M | RAEB | –3 | **Other/CTCL, erythroderma**  *Site:* abdomen, lumbar region, arms, palms and soles | NA | 0 (0%) survival  *Cause of death:* Two weeks after admission, the patient died of pneumonia and septic shock |
| Pinal-Fernandez, 2015 | 75, M | RAEB-1 | –24 | **Neutrophilic/Sweet syndrome H**  **Vasculitis/cutaneous polyarteritis nodosa**  *Site: anterior and lateral lower extremities* | 1.Prednisone 0.3–0.5 mg/kg/day, azathioprine and cyclophosphamide (poor response)  2.azacitidine and corticosteroids (0.2 mg/kg/day) (good response) | 1 (100%) survival  Remission |
| Pourmoussa, 2017 | 68, M | Unspecified | 0 | **Neutrophilic/Sweet syndrome**  *Site: bilateral lower extremities* | 1. Broad-spectrum antibiotics, blood products, Prednisone  2. high-dose methylprednisolone | 0 (0%) survival  *Cause of death:* MDS evolving into AML |
| Raj, 2007 | 74, M | Multilineage dysplasia | +12 | **Neutrophilic/Sweet syndrome**  *Site:* scalp, forehead, cheeks, neck; thighs. | 5-azacytidine 75 mg m2 subcutaneously for 7 days every 28 days. | 1 (100%) survival  Remission |
| Reina, 2013 | 70, M | RAEB-1 | –0.5 | **Panniculitis/Neutrophilic panniculitis**  *Site: extremities* | Glucocorticoids 0.5 mg/kg, indomethacin (150 mg/day) and potassium iodide - without improvement. RBC and bone marrow transplant. | 0 (0%) survival  *Cause of death:* AML |
| Reuss-Borst, 1993 | 56, F | Ring sideroblasts | +24 | **Neutrophilic/Sweet syndrome**  *Site:* *right thigh and left posterior iliac crest* | Cyclophosphamide (2 mg/kg/day). | 1 (100%) survival  Remission within 2 weeks |
| Saleh, 2017 | 50, F | Multilineage dysplasia | –5 | **Neutrophilic/Pyoderma gangrenosum**  *Site:* lower left leg circumferentially from ankle to knee. | IV (methylprednisolone and IVIG 5 days followed by oral prednisone 1 mg/kg (mild improvement) | 0 (0%) survival  *Cause of death:* Aspiration pneumonia 1 year later |
| Sargin, 2015 | 54, M | Single lineage dysplasia | 0 | **CTD/Systemic sclerosis**  *Site: face, fingers, gluteal region* | Metoclopramide, acetylsalicylic acid, methylprednisolone, bosentan, nipedipine, and azathioprine, RBC | 1 (100%) survival  NA |
| Schneider, 2006 | 68, M | Single lineage dysplasia | –36 | **Others/Lymphoma, pruritic papules andplaques**  *Site: chest* | 1. 3-week course of topical steroids  2. Short course of oral prednisolone | 1 (100%) survival  Partial remission but relapse 3-4 weeks after discontinuation |
| Shalaby, 2016 | 66, F | unspecified | +12 | **Neutrophilic/Sweet syndrome**  *Site:* *Cheeks and upper eyelid, L upper extremity; Lower extremities* | Oral prednisone 90mg OD and dapsone 5% gel. | 1 (100%) survival  NA |
| Takagi, 1998 | 35, M | RAEB | +58 | **Neutrophilic/PG**  *Site:* *upper and lower extremities* | Oral prednisolone 40mg | 1 (100%) survival  NA |
| Tomasini, 2000 | 68, M | RAEB | +3 | **Neutrophilic/Sweet syndrome**  *Site: chest, back, upper and lower extremities, face,lip* | Prednisolone (good initially, then unresponsive)  Frequent blood transfusions | NA  Worsening cytopenia |
| Tsuji, 2003 | 50, M | Unclassifiable | +3 | **CTD/Dermatomyositis**  *Site:* *face, neck, and forearms* | Oral prednisolone 60 mg per day | NA |
| Vazquez, 2001 | 72, F | Unspecified | +1 | **Other/ 1) Granuloma faciale**  **Neutrophilic/ 2) Sweet syndrome 3)PG**  *Site: 1) cheek, forehead, nose 2) back 3) leg* | 2) Prednisone 60 mg OD  3) Dapsone 100mg/day, lowered to 25mg/day,  Good response | 1 (100%) survival  Remission |
| Vera-Lastra, 2021 | 23, F | Unspecified | 0 | **Neutrophilic/Sweet syndrome**  *Site: upper inner quadrant of Right breast; left thigh* | NA | 0 (0%) survival  *Cause of death:* Progressed to AML (subtype M2), MOF |
| Wang, 2018 | 32, M | Single lineage dysplasia | NA | **Neutrophilic/Sweet syndrome**  *Site: face, trunk and limbs; tip of finger* | Ceftriaxone + levofloxacin + rifampicin + doxycycline for 6 months  Methylprednisolone 60 mg OD 10 months, weaned to 8mg OD PO | 1 (100%) survival  Remission |
| Watanabe, 1992 | 48, F | RAEB-2 | –48 | **Neutrophilic/Sweet syndrome**  *Site: Extensor aspect of right thigh; left forearm, right knee, and dorsum of left hand; limbs* | Prednisolone 30mg OD – improved. Weaning dose, discharged after 4 months' hospitalization. Varying doses of prednisolone over 2 years  Aspirin to prevent thrombosis. | 0 (0%) survival  *Cause of death:* generalised TB (2 years later) |
| Weed, 2017 | 72, F | Multilineage dysplasia | +60 | **Granulomatous/paraneoplastic granulomatous dermatosis**  *Site:* *posterior neck, upper and lower extremities, and abdomen* | EPO (unsuccessful), azacitidine for 3 years (worsening cytopenia)  Decitabine for 2 years. | 0 (0%) survival  *Cause of death:* sepsis, MOF, lactic acidosis |
| Xiao, 2007 | 53, M | Single lineage dysplasia | +132 | **Neutrophilic/Sweet syndrome**  *Site:* *left eye; bilateral face, neck and back.* | Prednisolone 45 mg OD and amoxycilline/sulbatamol 2.25 g BD  Weaning dose prednisolone | 1 (100%) survival  Remission |
| Yamamoto, 2001 | 58, M | Single lineage dysplasia | NA | **Other/Annular erythema**  *Site: back, face, hands* | NA | 0 (0%) survival  *Cause of death:* |
| Yang, 2011 | 46, M | RAEB-2 | –3 | **Neutrophilic/Pyoderma gangrenosum**  *Site: face, inguinal area* | 1. Oral pred 60mg OD + oral dapsone 100mg OD (resolved)  2. Amphotericin B, IV ceftriaxone, caspofungin 50mg | 0 (0%) survival  *Cause of death:* disseminated invasive aspergillosis, died 35 days after antifungals |
| Yates, 1987 | 54, M | Single lineage dysplasia | +10 | **Neutrophilic/PG**  *Site: forearm, hand, face, trunk* | IV antibiotics (improved cellulitis, PG worsened)  IV hydrocortisone / methyprednisolone and oral prednisolone. | 0 (0%) survival  *Cause of death:* AML. Died with pneumocystis pneumonia. |
| Yoneta, 2016 | 80, M | Unspecified | +36 | **Granulomatous/Disseminated granulomatous disease**  *Site: Head, neck, chest* | 1. Oral etretinate and topical steroids (improved the erythema, papules exacerbated.)  2. Oral tranilast improved) | 0 (0%) survival  *Cause of death:* AML, six months after the initiation of tranilast. |
| Yu, 2016 | 89, M | Single lineage dysplasia | NA | **Other/EPDS**  *Site: R parietal scalp* | Topical 0.1% tacrolimus ointment BD + topical mupirocin ointment | 1 (100%) survival  Remission |
| Case series |  |  |  |  |  |  |
| del Pozo, 2005 | 74, F  1/2 | Unclassified | *0* | ***Vasculitis/Leukocytoclastic vasculitis; 1*** | Blood transfusion  Topical and oral corticosteroid. | 0 (0%) survival  *Cause of death:* pneumonia with cardiac and respiratory failure |
| Jacobs, 1985 | 39, 58, 78, 60, 4M  4/6 | RAEB x 1, sideroblast x 1, unspecified x 2 | –2 to +12 | **Neutrophilic/PG: 4** | Prednisolone, RBCs | 0/3, 1 NA (0%) survival  *Cause of death: septicaemia (inc 1 x pneumonia)* |
| Kazakov, 2003 | 86, 71, 2M  2/3 | 2 unspecified | +7 to +11 | **Others/Nodules, ecchymosis: 2** | 1. Topical CS – partial remission 2. Thalidomide, radiotherapy, surgery – partial remission | 0 (0%) survival  *Cause of death: dead of disease at 4 – 14 months* |
| Evans, 2002 | 70M, 79M  2M  2/2 | 2 unspecified | –3 to  –12 | **Neutrophilic/Sweet syndrome: 2** | 1. Dapsone 150mg OD (controlled temporarily) / PO cyclophosphamdie 50mg OD more effective 2. Topical clobetasol propionate, pred daily | 2 (100%) survival  *Remission* |
| Luherne, 2021 | 1/2  70F | Single lineage | –3 | **Granulomatous/Disseminated cutaneous granulomatosis: 1** | Systemic steroids (10 mg/day) (rapid resolution)  Methotrexate (7.5 mg/week) Erythropoietin for anaemia | 0 (0%) survival  *Cause of death: infectious complications 3 years later* |
| Morioka, 1990 | 2/2  50M, 48F | RA, RAEB | –24 to +5 | **Neutrophilic/Sweet syndrome: 2** | GCS-F  Prednisolone | 0 (0%) survival  *Cause of death:*  *1.* *Developed AML 2.5 months later, died of SAH*  *2.TB* |
| Qian, 2015 | 2/2  35M 46M  2M | 2 Multilineage dysplasia | 0 | **Panniculitis/** **Neutrophilic panniculitis: 2** | Methylprednisolone (1 mg/kg) | 2 (100%) survival  *Good response* |
| Billstrom, 1995 | 6/82  4M2F  65, 60, 66, 71, 55 | 3 RA, 3 RAEB | 0 to +20 | **Neutrophilic/Sweet syndrome: 1**  **Vasculitis/Vasculitis: 4**  **Panniculitis/Panniculitis: 1** | NA | NA |
| Billings, 2004 | 2/2  80M 75M  2M | unspecified | +36 | **Neutrophilic/Sweet syndrome: 2** | NA | NA |
| Hamada, 2008 | 4/4  33M 50M, 31M, 23F  3M 1F | 3 RA, 1  ringed sideroblasts | NA | **Neutrophilic/neutrophilic dermatosis: 3**  **Other/Chronic pyoderma glutaele: 1** | NA | NA |
| Pagliuca, 1990 | 2/4  43, 25  1M 1F | RA, RAEB | 0 to +120 | **Vasculitis/Vasculitis: 2** | High dose steroids | 2 (100%) survival  *Some improvement* |
| Shimizu, 2016 | 5/239  3M 2F | unspecified | NA | **Panniculitis/erythema nodosum: 1**  **Neutrophilic/Sweet syndrome: 2**  **2 not mentioned** | NA | 4 (80%) survival  *Cause of death: 1 x unidentified cause* |
| Vestey, 1993 | 2/2  2M  66, 71 | 1 unspecified  1 RAEB-1 | –3.5 to –4 | **Granulomatous/granuloma annulare: 2** | Chemotherapy (*doxorubicin, cytosine, arabinoside and thioguanine)* x 1 (after AML) | 2 (100%) survival  1 x developed AML but achieved remission post chemotherapy |
| Weenig, 2004 | 1/4  62F | RAEB | NA | **Neutrophilic/Sweet syndrome: 1** | Prednisolone (improved) | 0 (0%) survival  *Cause of death: no information* |
| Horiguchi, 1998 | 3M  45-82 | 2 RA | 0 | **Neutrophilic/Sweet syndrome: 2** | Prednisolone 5-10mg OD and potassium iodide 900mg OD  Bethamethasone | 3 (100%) survival  *Improvement in skin* |
| Vignon-Pennamen, 1991 | 1/7  66  1F | 1 unspecified | NA | **Neutrophilic/Sweet syndrome: 1** | Topical steroids and prednisolone | 1 (100%) survival  Good response |
| Ghoufi, 2016 | 8/62  Gender NA | 8 unspecified | NA | **Neutrophilic/Sweet syndrome H: 7, N: 1** | High potent topical steroids and/or systemic steroids, hydroxychloroquine  colchicine, thalidomide , dapsone, NSAID, intravenous immunoglobulins with poor efficacy. | 7 (87.5%) survival H-SS  1 (12.5%) death N-SS  *Cause of death: 8 months after the diagnosis of H-SS and 2 years after onset of hematological disease* |
| Farah, 2010 | 10/157  11M 4F | NA | NA | **Neutrophilic/Sweet syndrome: 7**  **Vasculitis/Leukocytoclastic vasculitis: 2**  **Vasculitis/ Behçet's disease: 1** | NA | *Cause of death:*  *3/7 death in Sweet Syndrome:*  *2 x 4-20 months after AML transformation*  *1 x 36 months after without transformation* |
| Green, 1990 | 5/6  71, 72, 64, 63, 73  4M, 1F | RAEB x 2, RARS, RAEB, RA | NA | **Vasculitis/cutaneous vasculitis: 5** | Corticosteroid | NA |
| Pragliuca, 1990 | 2/4  43, 25  1M 1F | RAEB 1  Single lineage 1 | 0 to +120 | **Vasculitis/Vasculitis: 2** | Steroid | 1 (50%) survival  Cause of death: Multi-organ failure |
| Snyder, 2018 | 2/2  50, 73  2M | Single lineage 1 Unspecified 1 | –1 to  –48 | **Neutrophilic/Sweet syndrome H:** 2 | Azacytidine, prednisolone | 1 (50%) survival  1 x hospice care with MOF  1 under monitoring |

CTCL, cutaneous T cell lymphoma. G-CSF, Granulocyte colony-stimulating factor; H-SS, histiocytoid Sweet syndrome, IGD, interstitial granulomatous dermatitiw; IVIG, intravenous immunoglobulin; MOF, multiorgan failure; N-SS, neutrophilic Sweet syndrome; PG, pyoderma gangrenosum; RA, refractory anaemia; RARS, refractory anaemia with ring sideroblasts; RAEB, refractory anaemia with excess blasts; RAEB-1, refractory anaemia with excess blasts-1; RAEB-2, refractory anaemia with excess blasts-2; SAH, subarachnoid haemorrhage.

^a^Onset – in relation to MDS diagnosis

**Appedendix S1.** References of included studies

1. Antic D, Bogdanovic A, Perunicic Jovanovic M, Jovanovic J, Elezovic I. Myeloid sarcoma of the skin in a patient with myelodysplastic syndrome. Acta Dermatovenerol Croat. 2015;23(2):134-7. PMID: 26228825.
2. Arandes-Marcocci J, Iglesias-Sancho M, Pérez-Muñoz N, Ene GS, Setó-Torrent N, Garcia-Herrera A, Fernández-Figueras MT. Diffuse dermal angiomatosis as the first manifestation of myelodysplastic syndrome. Australas J Dermatol. 2021 Nov;62(4):529-530. doi: 10.1111/ajd.13698. Epub 2021 Aug 23. PMID: 34424548.
3. Ashida T, Mayama T, Higashishiba M, Kawanishi K, Miyatake J, Tatsumi Y, Kanamaru A. Successful reduced-intensity stem cell transplantation in a patient with myelodysplastic syndrome combined with Sweet's syndrome. Hematology. 2006 Jun;11(3):179-81. doi: 10.1080/10245330600667492. PMID: 17325958.
4. O’Donnell B.F., Williams H.C., Carr R. Myelodysplastic syndrome presenting as cutaneous vasculitis, Clinical and Experimental Dermatology, Volume 20, Issue 5, 1 September 1995, Pages 439–442, <https://doi.org/10.1111/j.1365-2230.1995.tb01369.x>
5. Balin SJ, Wetter DA, Kurtin PJ, Letendre L, Pittelkow MR. Myelodysplastic syndrome presenting as generalized granulomatous dermatitis. Arch Dermatol. 2011 Mar;147(3):331-5. doi: 10.1001/archdermatol.2011.39. PMID: 21422341.
6. Bhattacharjee P, Umar SA, Fatteh SM. Multiple eruptive dermatofibromas occurring in a patient with myelodysplastic syndrome. Acta Derm Venereol. 2005;85(3):270-1. doi: 10.1080/00015550410024517. PMID: 16040422.
7. Chamoun K, Loghavi S, Pemmaraju N, Konopleva M, Kroll M, Nguyen-Cao M, Hornbaker M, DiNardo CD, Kadia T, Jorgensen J, Andreeff M, Hu S, Benton CB. Early detection of transformation to BPDCN in a patient with MDS. Exp Hematol Oncol. 2018 Oct 6;7:26. doi: 10.1186/s40164-018-0117-6. PMID: 30323983; PMCID: PMC6174068.
8. Chen HC, Kao WY, Chang DM, Gao HW, Lai WY, Lai JH. Neutrophilic panniculitis with myelodysplastic syndromes presenting as pustulosis: case report and review of the literature. Am J Hematol. 2004 May;76(1):61-5. doi: 10.1002/ajh.20053. PMID: 15114599.
9. Choi HJ, Chang SE, Lee MW, Choi JH, Moon KC, Koh JK. A case of recurrent Sweet's syndrome in an 80-year-old man: a clue to an underlying malignancy. Int J Dermatol. 2006 Apr;45(4):457-9. doi: 10.1111/j.1365-4632.2004.02376.x. PMID: 16650178.
10. de Arruda Câmara VM, Morais JC, Portugal R, da Silva Carneiro SC, Ramos-e-Silva M. Cutaneous granulocytic sarcoma in myelodysplastic syndrome. J Cutan Pathol. 2008 Sep;35(9):876-9. doi: 10.1111/j.1600-0560.2007.00916.x. Epub 2008 May 20. PMID: 18494822.
11. Del Pozo, J., Martínez, W., Pazos, J.M., et al. Concurrent Sweet's syndrome and leukemia cutis in patients with myeloid disorders. International Journal of Dermatology. 2005. 44: 677-680. <https://doi.org/10.1111/j.1365-4632.2005.02037.x>
12. Delplanque M, Chasset F, Hirsch P, et al. Cutaneous lupus with Kikuchi disease-like inflammatory pattern associated with myelodysplastic syndrome. Rheumatology (Oxford). 2019 Mar 1;58(3):554-556. doi: 10.1093/rheumatology/key413. PMID: 30590766.
13. Farmakis D, Polymeropoulos E, Polonifi A, et al. Myelodysplastic syndrome associated with multiple autoimmune disorders. Clin Rheumatol. 2005 Aug;24(4):428-30. doi: 10.1007/s10067-004-1059-4. Epub 2005 Apr 13. PMID: 15827686.
14. Fein H, Adams BB. Myelodysplastic syndrome presenting as cutaneous purpura. Cutis. 2000 Jun;65(6):367-70. PMID: 10879305.
15. Goto A, Yamamoto S, Notoya A, Takada A, Mukai M. Pyoderma gangrenosum complicated with myelodysplastic syndrome followed by rapidly progressing pyothorax-associated lymphoma: a case report. Hokkaido Igaku Zasshi. 2006 Jul;81(4):261-4. PMID: 16892654.
16. Gubinelli E, Cocuroccia B, Fazio M, Annessi G, Girolomoni G. Papular neutrophilic dermatosis and erythema elevatum diutinum following erythropoietin therapy in a patient with myelodysplastic syndrome. Acta Derm Venereol. 2003;83(5):358-61. doi: 10.1080/00015550310003791. PMID: 14609104.
17. Hagiwara A, Fujimura T, Furudate S, Kambayashi Y, Numata Y, Haga T, Aiba S. Generalized granulomatous dermatitis accompanied by myelodysplastic syndrome. Acta Derm Venereol. 2014 Mar;94(2):223-4. doi: 10.2340/00015555-1656. PMID: 23817627.
18. Hattori H, Hoshida S, Yoneda S. Sweet's syndrome associated with recurrent fever in a patient with trisomy 8 myelodysplastic syndrome. Int J Hematol. 2003 May;77(4):383-6. doi: 10.1007/BF02982648. PMID: 12774928.
19. Hojo N, Hasegawa H, Iwamasa K, Hojo S, Fujita S. A case of Weber-Christian disease associated with myelodysplastic syndrome. Mod Rheumatol. 2004;14(1):73-6. doi: 10.1007/s10165-003-0270-5. PMID: 17028810.
20. Jacobs P, Palmer S, Gordon-Smith EC. Pyoderma gangrenosum in myelodysplasia and acute leukaemia. Postgrad Med J. 1985 Aug;61(718):689-94. doi: 10.1136/pgmj.61.718.689. PMID: 4034458; PMCID: PMC2418352.
21. Kakaletsis N, Kaiafa G, Savopoulos C, Iliadis F, Perifanis V, Tzalokostas V, Grekou A, Giannouli A, Hatzitolios AI. Initially lymphocytic Sweet's syndrome in male patients with myelodysplasia: a distinguished clinicopathological entity? Case report and systematic review of the literature. Acta Haematol. 2014;132(2):220-5. doi: 10.1159/000357933. PMID: 24714374.
22. Kamimura A, Yanagisawa H, Tsunemi Y, Kusano T, Arai E, Tsuchida T, Nakamura K. Normolipemic xanthomatized Sweet's syndrome: A variant of Sweet's syndrome with myelodysplastic syndrome. J Dermatol. 2021 May;48(5):695-698. doi: 10.1111/1346-8138.15781. Epub 2021 Feb 13. PMID: 33580905.
23. Kawakami T, Kimura S, Kato M, Mizoguchi M, Soma Y. Transforming growth factor-beta overexpression in cutaneous extramedullary hematopoiesis of a patient with myelodysplastic syndrome associated with myelofibrosis. J Am Acad Dermatol. 2008 Apr;58(4):703-6. doi: 10.1016/j.jaad.2007.10.489. PMID: 18342720.
24. Kazakov DV, Mentzel T, Burg G, Dummer R, Kempf W. Blastic natural killer-cell lymphoma of the skin associated with myelodysplastic syndrome or myelogenous leukaemia: a coincidence or more? Br J Dermatol. 2003 Oct;149(4):869-76. doi: 10.1046/j.1365-2133.2003.05639.x. PMID: 14616384.
25. Khodadad K, Sadeghipour A, Aghili N. Generalized neutrophilic dermatosis: a rare presentation of myelodysplastic syndrome. Indian J Cancer. 2005 Jan-Mar;42(1):57-9. doi: 10.4103/0019-509x.15102. PMID: 15805694.
26. Kim YJ, Yang HJ, Lee MW, Lee WJ. Cutaneous indicator of myelodysplastic syndrome: sudden bullous pyoderma gangrenosum. Jpn J Clin Oncol. 2020 Aug 4;50(8):958-959. doi: 10.1093/jjco/hyz207. PMID: 32129441.
27. Komiya I, Tanoue K, Kakinuma K, Kaneda M, Shinohara T, Kuriya S, Nomura T, Saito Y. Superoxide anion hyperproduction by neutrophils in a case of myelodysplastic syndrome. Association with Sweet's syndrome and interstitial pneumonia. Cancer. 1991 May 1;67(9):2337-41. doi: 10.1002/1097-0142(19910501)67:9<2337::aid-cncr2820670921>3.0.co;2-0. PMID: 1849446.
28. Lee SH, Kim JH, Park S, Won CY, Lee JH, Yi SY, Park HK, Chang SH, Jung H, Lee SS, Koo HK. Pulmonary Leukocytoclastic Vasculitis as an Initial Presentation of Myelodysplastic Syndrome. Tuberc Respir Dis (Seoul). 2016 Oct;79(4):302-306. doi: 10.4046/trd.2016.79.4.302. Epub 2016 Oct 5. PMID: 27790283; PMCID: PMC5077735.
29. Lee YY, Bee PC, Lee CK, Naiker M, Ismail R. Bullous pemphigoid in an elderly patient with myelodysplastic syndrome and refractory anemia coupled with excess of blast. Ann Dermatol. 2011 Dec;23(Suppl 3):S390-2. doi: 10.5021/ad.2011.23.S3.S390. Epub 2011 Dec 27. PMID: 22346286; PMCID: PMC3276805.
30. Lerman I, Richardson CT. Anti-TIF1gamma Antibody-Positive Dermatomyositis Associated with Myelodysplastic Syndrome: Response to Treatment. Cureus. 2019 Sep 26;11(9):e5775. doi: 10.7759/cureus.5775. PMID: 31723534; PMCID: PMC6825482.
31. Litvak D, Kirsner RS, Pakdaman NN, Federman DG. Pyoderma gangrenosum and myelodysplastic syndrome. South Med J. 2000 Sep;93(9):923-5. PMID: 11005358.
32. Martinelli S, Rigolin GM, Leo G, Gafà R, Lista E, Cibien F, Sofritti O, Daghia G, Cavazzini F, Cuneo A. Complete remission of Sweet's syndrome after azacytidine treatment for concomitant myelodysplastic syndrome. Int J Hematol. 2014;99(5):663-7. doi: 10.1007/s12185-014-1527-9. Epub 2014 Feb 20. PMID: 24554168.
33. Martínez-García M, Silvestre-Torner N, Aguilar-Martínez A, Burgos-Lázaro F. Multiple Xanthogranulomas in an Adult Patient with Myelodysplastic Syndrome. Case Rep Dermatol Med. 2020 Dec 7;2020:8826715. doi: 10.1155/2020/8826715. PMID: 33489387; PMCID: PMC7803178.
34. Marullo S, Dallot A, Cavelier-Balloy B, Valensi F, Clauvel JP. Subcutaneous eosinophilic necrosis associated with refractory anemia with an excess of myeloblasts. J Am Acad Dermatol. 1989 Feb;20(2 Pt 2):320-3. doi: 10.1016/s0190-9622(89)70038-4. PMID: 2915075.
35. Mégarbane, B., Bodemer, C., Valensi, F., Radford-Weiss, I., Fraitag, S., MacIntyre, E., Bletry, O., Varet, B. and Hermine, O. (2000), Association of acute neutrophilic dermatosis and myelodysplastic syndrome with (6; 9) chromosome translocation: a case report and review of the literature. British Journal of Dermatology, 143: 1322-1324. <https://doi.org/10.1046/j.1365-2133.2000.03912.x>
36. Mizes A, Khosravi H, Bordelon J, Kazlouskaya V, Karunamurthy A, Ho J, James A, Patton T. Sweet syndrome with pulmonary involvement in a patient with myelodysplastic syndrome. Dermatol Online J. 2020 Mar 15;26(3):13030/qt1n73f6k5. PMID: 32609450.
37. Morioka N, Otsuka F, Nogita T, Igisu K, Urabe A, Ishibashi Y. Neutrophilic dermatosis with myelodysplastic syndrome: nuclear segmentation anomalies of neutrophils in the skin lesion and in peripheral blood. J Am Acad Dermatol. 1990 Aug;23(2 Pt 1):247-9. doi: 10.1016/0190-9622(90)70206-w. PMID: 2212120.
38. Nakanishi T, Horikoshi H, Kusanagi Y, Yamamura T, Takahashi R, Kimura F, Itoh K. Refractory Dermatomyositis Complicated with Myelodysplastic Syndrome. Intern Med. 2015;54(19):2507-11. doi: 10.2169/internalmedicine.54.4762. Epub 2015 Oct 1. PMID: 26424313.
39. Namba Y, Koizumi H, Nakamura H, Tarumi T, Sawada K, Ohkawara A. Specific cutaneous lesions of the scalp in myelodysplastic syndrome with deletion of 20q. J Dermatol. 1999 Apr;26(4):220-4. doi: 10.1111/j.1346-8138.1999.tb03460.x. PMID: 10343466.
40. Nawata T, Kubo M, Okuda S, Omoto M, Yujiri T, Kanda T, Yano M. Successful treatment with intravenous cyclophosphamide for anti-melanoma differentiation-associated gene 5 antibody-positive dermatomyositis associated with myelodysplastic syndrome. Scand J Rheumatol. 2017 Nov;46(6):496-498. doi: 10.1080/03009742.2016.1253770. Epub 2016 Dec 9. PMID: 27931156.
41. Nifosì G, Sbolli G, Ferrari B, Berte' R, Vallisa D, Civardi G, Cavanna L. Sweet's syndrome associated with monosomy 7 myelodysplastic syndrome. Eur J Intern Med. 2001 Jul;12(4):380-383. doi: 10.1016/s0953-6205(01)00137-6. PMID: 11395304.
42. Nishie W, Kimura T, Kanagawa M. Sweet's syndrome evolved from recurrent erythema nodosum in a patient with myelodysplastic syndrome. J Dermatol. 2002 Feb;29(2):91-5. doi: 10.1111/j.1346-8138.2002.tb00172.x. PMID: 11890302.
43. Nizery-Guermeur C, Le Gall-Ianotto C, Brenaut E, Couturier MA, Talagas M, Andrieu-Key S, Guillerm G, Misery L, Karam A. Cutaneous granulocytic sarcoma and Koebner phenomenon in a context of myelodysplastic syndrome. JAAD Case Rep. 2015 Jun 17;1(4):207-11. doi: 10.1016/j.jdcr.2015.04.012. PMID: 27051731; PMCID: PMC4808728.
44. Palterer B, Vitiello G, Cammelli D. First report of anti-TIF1γ dermatomyositis in a patient with myelodysplastic syndrome. Reumatismo. 2017 Aug 3;69(2):75-77. doi: 10.4081/reumatismo.2017.923. PMID: 28776361.
45. Papaioannou M, Sapalidis K, Kotoula V. Alleviation of myelodysplastic syndrome-associated skin rush after treatment with decitabine. Eur J Haematol. 2008 Dec;81(6):489-90. doi: 10.1111/j.1600-0609.2008.01135.x. Epub 2008 Aug 6. PMID: 18691250.
46. Patsinakidis N, Susok L, Hessam S, Othlinghaus N, Möllenhoff K, Stücker M, Altmeyer P, Kreuter A. Interstitial granulomatous dermatitis associated with myelodysplastic syndrome - complete clearance under therapy with 5-azacytidine. Acta Derm Venereol. 2014 Nov;94(6):725-6. doi: 10.2340/00015555-1827. PMID: 24573709.
47. Peñas PF, Jones-Caballero M, Daudén E, Fraga J, García-Díez A. Cutaneous T-cell lymphoma and myelodysplastic syndrome. J Am Acad Dermatol. 1994 Dec;31(6):1065-7. doi: 10.1016/s0190-9622(09)80090-x. PMID: 7962764.
48. Pinal-Fernandez I, Ferrer Fabrega B, Ramentol Sintas M, Solans Laque R. Histiocytoid Sweet syndrome and cutaneous polyarteritis nodosa secondary to myelodysplastic syndrome. Int J Rheum Dis. 2013 Dec;16(6):777-9. doi: 10.1111/1756-185X.12103. Epub 2013 Jun 3. PMID: 24382288.
49. Pourmoussa A, Kwan K. An Unlikely Rapid Transformation of Myelodysplastic Syndrome to Acute Leukemia: A Case Report. Perm J. 2017;21:16-091. doi: 10.7812/TPP/16-091. PMID: 28488979; PMCID: PMC5424583.
50. Qian L, Shen J, Cen J, Yin W, Ma Y. Myelodysplastic syndrome with neutrophilic panniculitis: A report of two cases and a literature review. Oncol Lett. 2015 Apr;9(4):1954-1956. doi: 10.3892/ol.2015.2932. Epub 2015 Feb 4. PMID: 25789075; PMCID: PMC4356265.
51. Raj K, Ho A, Creamer JD, du Vivier AW, Salisbury JR, Mufti GJ. Complete response of deep neutrophilic dermatosis associated with myelodysplastic syndrome to 5-azacytidine. Br J Dermatol. 2007 May;156(5):1039-41. doi: 10.1111/j.1365-2133.2007.07831.x. Epub 2007 Apr 4. PMID: 17408390.
52. Reina D, Cerdà D, Roig D, Fíguls R, Villegas ML, Corominas H. Sweet syndrome associated with myelodysplastic syndrome: report of a case. Review of the literature. Reumatol Clin. 2013 Jul-Aug;9(4):246-7. English, Spanish. doi: 10.1016/j.reuma.2012.01.014. Epub 2012 Jun 30. PMID: 22749728.
53. Reuss-Borst MA, Pawelec G, Saal JG, Horny HP, Müller CA, Waller HD. Sweet's syndrome associated with myelodysplasia: possible role of cytokines in the pathogenesis of the disease. Br J Haematol. 1993 Jun;84(2):356-8. doi: 10.1111/j.1365-2141.1993.tb03083.x. PMID: 7691149.
54. Saleh MFM, Saunthararajah Y. Severe pyoderma gangrenosum caused by myelodysplastic syndrome successfully treated with decitabine administered by a noncytotoxic regimen. Clin Case Rep. 2017 Oct 31;5(12):2025-2027. doi: 10.1002/ccr3.1221. PMID: 29225849; PMCID: PMC5715603.
55. Sargın G, Şentürk T, Yavaşoğlu İ. Refractory anemia in systemic sclerosis: myelodisplastic syndrome. Eur J Rheumatol. 2015 Sep;2(3):120-121. doi: 10.5152/eurjrheum.2015.0100. Epub 2015 Mar 31. PMID: 27708945; PMCID: PMC5047254.
56. Schneider LA, Schmid M, Staib G, Weiss T, Scharffetter-Kochanek K, Weber L. Cutaneous infiltrations can herald an inapparent myelodysplastic syndrome. Acta Derm Venereol. 2006;86(2):172-3. doi: 10.2340/00015555-0025. PMID: 16648929.
57. Shalaby MM, Riahi RR, Rosen LB, Soine EJ. Histiocytoid Sweet's syndrome in a patient with myelodsyplastic syndrome: report and review of the literature. Dermatol Pract Concept. 2016 Jan 31;6(1):9-13. doi: 10.5826/dpc.0601a04. PMID: 26937301; PMCID: PMC4758439.
58. Takagi S, Ohsaka A, Taguchi H, Kusama H, Matsuoka T. Pyoderma gangrenosum following cytosine arabinoside, aclarubicin and granulocyte colony-stimulating factor combination therapy in myelodysplastic syndrome. Intern Med. 1998 Mar;37(3):316-9. doi: 10.2169/internalmedicine.37.316. PMID: 9617871.
59. Ten Oever J, Kuijper PH, Kuijpers AL, Dercksen MW, Vreugdenhil G. Complete remission of MDS RAEB following immunosuppressive treatment in a patient with Sweet's syndrome. Neth J Med. 2009 Sep;67(8):347-50. PMID: 19767665.
60. Tomasini C, Aloi F, Osella-Abate S, Dapavo P, Pippione M. Immature myeloid precursors in chronic neutrophilic dermatosis associated with myelodysplastic syndrome. Am J Dermatopathol. 2000 Oct;22(5):429-33. doi: 10.1097/00000372-200010000-00008. PMID: 11048979.
61. Tsuji G, Maekawa S, Saigo K, Nobuhara Y, Nakamura T, Kawano S, Koshiba M, Asahara S, Chinzei T, Kumagai S. Dermatomyositis and myelodysplastic syndrome with myelofibrosis responding to methotrexate therapy. Am J Hematol. 2003 Nov;74(3):175-8. doi: 10.1002/ajh.10430. PMID: 14587044.
62. Vázquez García J, Almagro Sánchez M, Fonseca Capdevila E. Multiple neutrophilic dermatoses in myelodysplastic syndrome. Clin Exp Dermatol. 2001 Jul;26(5):398-401. doi: 10.1046/j.1365-2230.2001.00844.x. PMID: 11488825.
63. Vera-Lastra O, Olvera-Acevedo A, Pulido-Díaz N, Quintal-Ramírez MJ, Ordoñez-González I, Cimé-Aké E, Cruz-Domínguez MP, Medina G. Transformation of a myelodysplastic syndrome to acute myeloid leukemia and concurrent necrotizing sweet syndrome. Dermatol Reports. 2021 Mar 17;13(1):9017. doi: 10.4081/dr.2021.9017. PMID: 33824709; PMCID: PMC8018258.
64. Wang W, Lu X, Li C, Ri MJ, Cui W. A man with recurrent fever, arthritis, and rashes-brucellosis? A case report. BMC Infect Dis. 2020 Jan 7;20(1):18. doi: 10.1186/s12879-019-4746-0. PMID: 31910802; PMCID: PMC6947870.
65. Watanabe R, Iijima M, Otsuka F. A case of neutrophilic dermatosis (ND) complicated by cryofibrinogenemia (CFGN) and myelodysplastic syndrome (MDS). J Dermatol. 1992 Mar;19(3):181-5. doi: 10.1111/j.1346-8138.1992.tb03203.x. PMID: 1640024.
66. Weed J, Ko C, Stahl M, Much M, Witt D, Zeidan AM, Leventhal J. Reactive granulomatous dermatitis presenting as subcutaneous nodules and cords in a patient with advanced myelodysplastic syndrome. Ann Hematol. 2017 Jun;96(6):1037-1039. doi: 10.1007/s00277-017-2954-5. Epub 2017 Feb 20. PMID: 28220192.
67. Xiao T, He CD, Gao XH, Chen HD. Sweet's syndrome associated with skin methicillin-resistant Staphylococcus epidermidis infection. J Dermatol. 2007 Apr;34(4):258-61. doi: 10.1111/j.1346-8138.2007.00264.x. PMID: 17352724.
68. Yamamoto T, Soejima K, Yokozeki H, Koyano T, Katayama I, Nishioka K. Unusual annular erythema associated with myelodysplastic syndrome. Dermatology. 2001;202(1):70-2. doi: 10.1159/000051592. PMID: 11244236.
69. Yang CC, Hsu PC, Cheng CW, Lee MH. Coexistence of fatal disseminated invasive aspergillosis and pyoderma gangrenosum: a case report. Med Princ Pract. 2011;20(4):380-3. doi: 10.1159/000324802. Epub 2011 May 11. PMID: 21577002.
70. Yates P, Corbett G, Stockdill G. Pyoderma gangrenosum and myelodysplasia. Clin Lab Haematol. 1987;9(4):425-8. doi: 10.1111/j.1365-2257.1987.tb00582.x. PMID: 3481693.
71. Yoneta K, Fujimoto N, Teramura K, Takayama S, Tanaka T. Disseminated granulomatous skin lesions associated with myelodysplastic syndrome treated successfully with tranilast: a case report and review of the literature. Eur J Dermatol. 2016 Aug 1;26(4):398-400. doi: 10.1684/ejd.2016.2812. PMID: 27229541.
72. Yu X, Liu Y, Ozukum M, Song Z. Erosive pustular dermatosis of the scalp associated with myelodysplastic syndrome. J Eur Acad Dermatol Venereol. 2016 Feb;30(2):380-1. doi: 10.1111/jdv.12812. Epub 2014 Nov 11. PMID: 25388746.
73. Evans AV, Sabroe RA, Liddell K, Russell-Jones R. Lymphocytic infiltrates as a presenting feature of Sweet's syndrome with myelodysplasia and response to cyclophosphamide. Br J Dermatol. 2002 Jun;146(6):1087-90. doi: 10.1046/j.1365-2133.2002.04701.x. PMID: 12072085.
74. Luherne C, Menguy S, Seneschal J, Pham-Ledard A, Beylot-Barry M. Disseminated cutaneous granulomatosis as a manifestation of myelodysplastic syndrome. Int J Dermatol. 2021 May;60(5):628-630. doi: 10.1111/ijd.15343. Epub 2020 Nov 28. PMID: 33247841.
75. Billström R, Johansson H, Johansson B, Mitelman F. Immune-mediated complications in patients with myelodysplastic syndromes--clinical and cytogenetic features. Eur J Haematol. 1995 Jul;55(1):42-8. doi: 10.1111/j.1600-0609.1995.tb00231.x. PMID: 7615049.
76. Billings SD, Hans CP, Schapiro BL, Martin RW 3rd, Fivenson D, Fruland JE, Moores WB, Cotton J. Langerhans cell histiocytosis associated with myelodysplastic syndrome in adults. J Cutan Pathol. 2006 Feb;33(2):171-4. doi: 10.1111/j.0303-6987.2006.00299.x. PMID: 16420314.
77. Hamada T, Matsuura H, Oono T, Morizane S, Yamasaki O, Asagoe K, Yamamoto T, Tsuji K, Iwatsuki K. Karyotypic analysis of bone marrow cells in pyodermic lesions associated with myelodysplastic syndrome. Arch Dermatol. 2008 May;144(5):643-8. doi: 10.1001/archderm.144.5.643. PMID: 18490591.
78. Shimizu J, Oka H, Yamano Y, Yudoh K, Suzuki N. Cutaneous manifestations of patients with relapsing polychondritis: an association with extracutaneous complications. Clin Rheumatol. 2016 Mar;35(3):781-3. doi: 10.1007/s10067-015-3160-2. Epub 2016 Jan 16. PMID: 26780448.
79. Vestey JP, Turner M, Biddlestone L, McLaren K, Goulden N, Hunter JA. Disseminated cutaneous granulomatous eruptions associated with myelodysplastic syndrome and acute myeloid leukaemia. Clin Exp Dermatol. 1993 Nov;18(6):559-63. doi: 10.1111/j.1365-2230.1993.tb01031.x. PMID: 8252798.
80. Weenig RH, Bruce AJ, McEvoy MT, Gibson LE, Davis MD. Neutrophilic dermatosis of the hands: four new cases and review of the literature. Int J Dermatol. 2004 Feb;43(2):95-102. doi: 10.1111/j.1365-4632.2004.01845.x. PMID: 15125498.
81. Horiguchi Y, Lee SG, Matsumoto I, Arima N, Fujii H, Ohnuma Y, Imamura S. Abscess-forming neutrophilic dermatosis: report of three cases associated with hemopathies. Dermatology. 1998;197(2):174-7. doi: 10.1159/000017993. PMID: 9732170.
82. Kakaletsis N, Kaiafa G, Savopoulos C, Iliadis F, Perifanis V, Tzalokostas V, Grekou A, Giannouli A, Hatzitolios AI. Initially lymphocytic Sweet's syndrome in male patients with myelodysplasia: a distinguished clinicopathological entity? Case report and systematic review of the literature. Acta Haematol. 2014;132(2):220-5. doi: 10.1159/000357933. PMID: 24714374.
83. Vignon-Pennamen MD, Wallach D. Cutaneous manifestations of neutrophilic disease. A study of seven cases. Dermatologica. 1991;183(4):255-64. doi: 10.1159/000247696. PMID: 1809587.
84. Farah C, Bulai Livideanu C, Jegu J, Paul C, Viraben R, Lamant L, Delavigne K, Adoue D, Laurent G, Beyne Rauzy O. Prevalence and prognostic value of cutaneous manifestations in patients with myelodysplastic syndrome. J Eur Acad Dermatol Venereol. 2010 Oct;24(10):1171-5. doi: 10.1111/j.1468-3083.2010.03614.x. PMID: 20202054.
85. Green AR, Shuttleworth D, Bowen DT, Bentley DP. Cutaneous vasculitis in patients with myelodysplasia. Br J Haematol. 1990 Mar;74(3):364-5. doi: 10.1111/j.1365-2141.1990.tb02597.x. PMID: 2278546.
86. Snyder R, Libby T, Raciti P, Amin B, Jacobson M, Rakheja D, Fleming K, Bartenstein M, Zhu C, Goel S, Verma AK, Shastri A. Myelodysplastic Syndrome and Sweet's Syndrome Are Associated with a Mutation in Isocitrate Dehydrogenase 1. Anticancer Res. 2018 Apr;38(4):2201-2205. doi: 10.21873/anticanres.12462. PMID: 29599340.
87. Ogawa H, Kuroda T, Inada M, Yamamoto M, Enomoto H, Kishima Y, Yoshida K, Ito H, Ogawa H, Nakamura H. Intestinal Behçet's disease associated with myelodysplastic syndrome with chromosomal trisomy 8--a report of two cases and a review of the literature. Hepatogastroenterology. 2001 Mar-Apr;48(38):416-20. PMID: 11379321.
88. Ghoufi L, Ortonne N, Ingen-Housz-Oro S, Barhoumi W, Begon E, Haioun C, Pautas C, Beckerich F, Robin C, Wolkenstein P, Cordonnier C, Chosidow O, Toma A. Histiocytoid Sweet Syndrome Is More Frequently Associated With Myelodysplastic Syndromes Than the Classical Neutrophilic Variant: A Comparative Series of 62 Patients. Medicine (Baltimore). 2016 Apr;95(15):e3033. doi: 10.1097/MD.0000000000003033. PMID: 27082547; PMCID: PMC4839791.
